# Dietary Patterns, Foods, and Nutrients to Ameliorate Non-Alcoholic Fatty Liver Disease: A Scoping Review

**DOI:** 10.3390/nu15183987

**Published:** 2023-09-14

**Authors:** Sofía Montemayor, Silvia García, Margalida Monserrat-Mesquida, Josep A. Tur, Cristina Bouzas

**Affiliations:** 1 Research Group on Community Nutrition & Oxidative Stress, University of the Balearic Islands-IUNICS, 07122 Palma, Spaincristina.bouzas@uib.es (C.B.); 2 Health Research Institute of Balearic Islands (IdISBa), 07120 Palma, Spain; 3 CIBEROBN (Physiopathology of Obesity and Nutrition), Instituto de Salud Carlos III, 28029 Madrid, Spain

**Keywords:** NAFLD, nutrients, food, dietary guidelines, dietary recommendations, liver steatosis

## Abstract

Background: Non-alcoholic fatty liver disease (NAFLD) is the most common cause of chronic liver disease without pharmacological treatment yet. There is also a lack of specific dietary recommendations and strategies to treat the negative health impacts derived from NAFLD. Objective: This scoping review aimed to compile dietary patterns, foods, and nutrients to ameliorate NAFLD. Methods: A literature search was performed through MEDLINE, Scopus, Web of Science, and Google Scholar. Results: Several guidelines are available through the literature. Hypocaloric Mediterranean diet is the most accepted dietary pattern to tackle NAFLD. Coffee consumption (sugar free) may have a protective effect for NAFLD. Microbiota also plays a role in NAFLD; hence, fibre intake should be guaranteed. Conclusions: A high-quality diet could improve liver steatosis. Weight loss through hypocaloric diet together with physical activity and limited sugar intake are good strategies for managing NAFLD. Specific dietary recommendations and a Mediterranean plate have been proposed to ameliorate NAFLD.

## 1. Introduction

The most usual cause of chronic liver disease worldwide is non-alcoholic fatty liver disease (NAFLD), with a worldwide prevalence of 20–30% of the adult population worldwide in 2017 [1], and an overall prevalence of 32.4% in 2022, which continues rising globally [2]. Non-alcoholic steatohepatitis (NASH) is the major contributor to the advancement of cirrhosis and hepatocellular carcinoma [3,4], which is the second most important cause of lost life years among all cancers [5].

NAFLD causes ongoing medical expenses, financial losses, and a decline in the quality of life [6,7,8]. It is also associated with obesity, metabolic syndrome (MetS) [9], type 2 diabetes mellitus (T2DM) [10], cardiovascular diseases (CVD) [11], hypertension, and chronic kidney disease (CKD) [12]. A proportion of cases has a normal body mass index (BMI), which is known as “non-obese NAFLD” or “lean NAFLD” [13]. Worldwide prevalence of non-obese NAFLD is growing substantially [14]. Lean NAFLD subjects showed similar complications and comorbidities to their obese counterparts, such as T2DM, cardiovascular diseases, and hepatocellular carcinoma [15,16]. Then, NAFLD is a major threat globally.

The recommended NAFLD/NASH treatment is changes in lifestyle (i.e., increasing exercise) [17]. Even in the absence of obesity, poor nutritional composition may put a person at risk for NAFLD [18,19]. The significance of weight loss (WL) is emphasized in these therapies, with a goal of a 7–10% decrease in body weight (BW) obtained by a hypocaloric diet (deficit of 500–1000 kcal/d) and/or physical activity. However, precise nutritional advice is inconsistent, and weight loss and maintenance remain a significant challenge for many people. The Mediterranean diet (MedDiet) is notably mentioned by the joint action of the European Association for the Study of the Liver (EASL)–the European Association for the Study of Diabetes (EASD)–the European Association for the Study of Obesity (EASO), by the European Society for Clinical Nutrition and Metabolism (ESPEN), and by the Asian Pacific Association for the Study of the Liver (APASL) as being advantageous for NAFLD patients [17,20,21,22]. Modifying dietary composition, with or without calorie restriction, may be a useful and long-lasting method of managing NAFLD [23,24]. The macronutrient proportion of the diet, particularly high intakes of carbohydrates, simple sugars, fats, proteins, and low fibre intake may be linked to the NAFLD risk, independent of excessive energy intake. The intake of certain nutrients may influence this disease, but its mechanism is not absolutely understood yet [23].

The aim of this scoping review is to compile dietary patterns, foods, and nutrients to ameliorate NAFLD.

## 2. Review Methodology

The relevant information on dietary treatment, weight loss strategies and dietary guidelines to ameliorate NAFLD was obtained from a literature search conducted between January and July 2023 through MEDLINE (via PubMed), Scopus, Web of Science and Google Scholar. MeSH terms used for the search were “non-alcoholic fatty liver disease”, “diet/dietary treatment”, “dietary guideline”, “weight loss strategy”. The most relevant data were summarized, and the results are presented in the following sections. Only papers written in English and Spanish were considered. A publication date filter was not applied, as the study of dietary treatment for NAFLD is quite recent.

## 3. Current Dietary Guidelines

Dietary guidelines are an important tool to address NAFLD based on a target population; however, they are unspecific regarding dietary recommendations for NAFLD patients. Scientific associations [17,20,21,22] highlighted the importance of weight loss targeting 7–10% reduction in body weight achieved by hypocaloric diets (energy deficit of 500–1000 kcal/day) and/or physical activity to promote a caloric deficit. EASL–EASD–EASO and APASL recommended the exclusion of processed food and added fructose. There is low evidence supporting dietary composition. There are no recommendations for macronutrients, but the join action of the European Association for the Study of the Liver (EASL)–the European Association for the Study of Diabetes (EASD)–the European Association for the Study of Obesity (EASO), the European Society for Clinical Nutrition and Metabolism (ESPEN), and the Asian Pacific Association for the Study of the Liver (APASL) mentioned that the MedDiet is useful to reduce liver fat and improve NAFLD. Table 1 shows the guidelines from the scientific associations that were made for different target populations.

## 4. Diet Impact in NAFLD, Gut Microbiota, and Macronutrients

Independently of weight loss and energy intake, a healthy diet is effective in decreasing the liver fat contents and thus protecting the liver from cardiometabolic morbidity and mortality [24]. Research showed that foods considered to be beneficial to prevent and avoid progression of NAFLD are fruits and vegetables, whole grain cereals, fatty fish (mainly rich in omega 3), and extra virgin olive oil [25]. It was pointed out that there is evidence that detrimental dietary composition predisposes an individual to NAFLD, even without obesity [19]. The foods considered NAFLD promoters are red meat and processed meats, soda, processed foods, cakes, and biscuits [24]. Diet directly impacts the process of de novo lipogenesis, in which hepatocytes transform the excess of carbohydrates, especially fructose, into fatty acids [26]. Daily intake of fructose is linked to high fibrosis, which is mainly due to industrial fructose and not to fruit-derived fructose [27]. This effect could be mediated by hepatic ATP depletion. Sweetened beverages containing fructose, sucrose, and/or high-fructose corn syrup are associated with an increased risk of developing steatosis and NASH, especially in overweight and obese people [28]. A cross-sectional study found that high consumption of red and/or processed meat was related to insulin resistance and NAFLD [29]. High animal protein intake was related to NAFLD in overweight patients [30]. Diet promotes NASH by modulating hepatic triglyceride accumulation and antioxidant activity by means of changes in postprandial triglyceride metabolism and insulin sensitivity [18,31]. 

Diet may prevent or trigger liver lipid accumulation by influencing the relationship between liver, adipose tissue, and gut, regardless of energy intake. For instance, formulations of high-fructose diets induced distinct changes in intestine microbiota. It has been reported that a high-fructose corn syrup reduced butyrate-producing bacteria and the Firmicutes/Bacteroidetes ratio linked to the metabolic syndrome pathogenesis [32,33], but high-fructose diet from fruits induced an opposite shift [34]. 

The macronutrient composition was related to NAFLD/NASH development [35]. Regarding carbohydrates, it was established that fructose is the primary carbohydrate determinant in hepatic fat formation. Fructose has a detrimental metabolic impact promoting de novo lipogenesis (DNL) and liver fat storage [36]. For instance, the MedDiet is characterized by low carbohydrate intake (low-fat diet: 40% calories vs. 50–60%), with low sugars and refined carbohydrates [37]. According to a previous study, 15% of liver fat is obtained from diet, and this percentage may rise if dietary fat consumption exceeds 30% of daily energy needs [38]. Saturated fatty acids impair phospholipid metabolism and increase insulin resistance by promoting mitochondrial dysfunction, which modifies respiratory chain activity and ATP homeostasis, increases reactive oxygen species (ROS) synthesis, and induces apoptosis [39]. The literature shows controversial and scarce data on the dietary protein effects in NAFLD patients. Animal models suggest that dietary proteins have benefits for glucose metabolism. In population studies, animal protein-rich diets are linked to an increased risk of NAFLD development, especially in patients who are overweight [40]. Another risk factor for the severity of NAFLD is the presence of sarcopenia and obesity and insulin resistance and inflammation, which are increased in the coexistence of fat accumulation and muscle loss, leading to atherosclerotic damage and severe liver disease (NASH and advanced fibrosis) [41].

## 5. Nutrition, Genetics, and NAFLD

Diet even impacts the DNA homeostasis where calcium, folate, nicotinic acid, vitamin E, and retinol are cofactors of essential enzymes in the DNA synthesis and repair [42], whereas others such as the trans fatty acids induce DNA damage [43]. In NAFLD, telomeres shortening is typical [44], but vegetables, fibre, and omega-3 fatty acids prevent it, which was significantly associated with decreases in low-density lipoprotein (LDL) cholesterol and psychological distress, as well as the anti-inflammatory contents of the diet [45], suggesting the role of these factors in telomere biology maintenance and potentially impacting overall health status. Saturated fatty acids, processed meat, and carbohydrates with a high glycaemic index favour it [46,47].

A major determinant of liver fat contents and the NAFLD progression is the patatin-like phospholipase domain-containing 3 (PNPLA3). It increases a mutated protein with impaired lipase activity on hepatocyte lipid droplets, favouring the accumulation of triglycerides in the liver [48,49]. Acquired variables such as diet, and especially carbohydrate-rich diets, can cause the protein encoding by the mutant gene [50]. It is also influenced by fatty acids, particularly *n*-6/*n*-3 PUFA [51]. Another important genetic determinant is the variant of the transmembrane 6 superfamily member 2 gene (TM6SF2), which predisposes an individual to increase liver fat content due to the retention of lipids and the impairment in the liver the release of very low-density lipoprotein (VLDL) [52]. In comparison to the wild types, carriers of the mutant TM6SF2 allele improved fasting and postprandial lipid profiles, which decrease circulating atherogenic lipoproteins even after a high fat challenge [53]. It is suggested that diet may modulate the epigenetic modifications, affecting the liver lipid metabolism, mitochondrial function, oxidative stress, and insulin resistance [54,55]. DNA methylation is considered a crucial step in the development of NASH and the metabolism of triglycerides, and it may be increased by dietary deficiencies in choline, betaine, B_12_, and folate, where the deficiencies encourage the overexpression of genes involved in the fatty acid production, which in turn promotes the synthesis of hepatic triglycerides [56]. 

## 6. Western Diet vs. Mediterranean Diet

A dietary pattern represents the overall combination of foods usually consumed, together with effects on health [57]. Studies showed that people following a high-fat Western dietary pattern were at an increased risk of developing NAFLD and its severity, while people following a MedDiet were associated with amelioration in hepatic steatosis and a lower rate of NAFLD [57,58,59]. The Western diet has a strong positive association with weight gain, insulin resistance, and NAFLD [59,60,61]. The NAFLD level found in Australian adolescents was prospectively linked to the Western dietary pattern, independently of family income, physical activity, and sedentary behaviour [62]. This dietary pattern lacks a precise definition; nevertheless, it can be generally characterized by densely caloric food, high animal protein, low fruit and vegetable contents, overeating [63], refined grains, red and processed meats, and soft drinks [64]. Recent studies showed the relation between higher intake of red and processed meats and NAFLD [30,65,66,67]. When they were consumed in excess, even for a short time such as one week, diets high in SFAs and high in fructose increased hepatic steatosis [68]. Lifestyle linked to Western diets included physical inactivity, usual snacking, high chronic psychological stress, smoking, environmental pollution, insufficient sleep, and avoidance of sun exposure [69,70]. NAFLD is related to short sleep duration, poor sleep quality [71], together with a trend for obstructive sleep apnoea syndrome [72]. A recent study in China in around 90,000 adults found that long-term exposure to environmental air pollution may increase the risk of MAFLD, particularly in men, smokers, alcohol drinkers, people who eat high-fat diets, and people who have central obesity. A study with rats showed that chronic exposure to ultraviolet radiation may be a good complementary therapy in the development of NAFLD [73].

On the contrary, the MedDiet is a plant-rich food diet, with olive oil being the highest source of added fats, high or moderate consumption of fish and shellfish, moderate consumption of eggs, poultry, and dairy products (mainly as cheese and yogurt), low consumption of meat, and moderate intake of alcohol (mainly wine with meals). This diet is rich in monounsaturated fatty acids (MUFAs) mainly from olive oil and olives, includes low-fat dairy products, and encourages low consumption of red meat. It has proven benefits to prevent cardiovascular diseases, hypertension, hypercholesterolemia, and obesity [24,74]. Several dietary approaches could be used to treat NAFLD [75], but the MedDiet was found especially helpful to improve the NAFLD. Cross-sectional, longitudinal studies and clinical trials showed a lower probability of NASH in patients who adhered to the MedDiet [76,77,78]. A 6-month nutritional counselling study showed that adherence to the MedDiet was effective in improving disease-specific traits, including liver imaging, liver fibrosis score, inflammatory/oxidative biomarkers, and indices of glycaemic status in nonfibrotic NAFLD patients [23]. A clinical trial conducted in Spain among NAFLD and MetS patients showed that an increase in MedDiet adherence over 6 months was related to better outcomes in terms of body weight, waist circumference, and BMI, but also in blood pressure (both systolic and diastolic), and to a decrease in intrahepatic fat content. In other words, an increase in MedDiet adherence ameliorated intrahepatic fat content after 6 months of treatment [76]. The MedDiet has been shown to reduce liver fat and to improve hepatic insulin sensitivity independent of exercise and weight loss [77]. High adherence to the MedDiet was highly associated with low insulin resistance level, alanine aminotransferase (ALT), and NAFLD severity [17]. Similarly, high adherence to the MedDiet was found to be associated with low likelihood of high-grade steatosis and the presence of steatohepatitis [78]. The positive effect of the MedDiet on liver inflammation and fibrosis was demonstrated in few observational studies and in small population studies. However, the European Association for the Study of the Liver (EASL), the European Association for the Study of Diabetes (EASD) and the European Association for the Study of Obesity (EASO) recommended the MedDiet for the treatment of NAFLD based on to this moderate-quality evidence in Europe [17]. The MedDiet has been shown to reduce hepatic fat and to improve hepatic insulin sensitivity even without weight loss in patients with NAFLD. Adherence to the MedDiet has also been associated with less advanced NAFLD and low risk of metabolic syndrome in NAFLD patients [74]. The MedDiet also encourages a high-quality lifestyle, including adequate rest periods, physical activity, and sun exposure, which showed positive effect on the NAFLD course [79]. The MedDiet Foundation, in collaboration with the Forum on Mediterranean Food Cultures, has started a process to gather scientific input from international specialists to reach a consensus position on a reviewed description of the MedDiet pyramid [79].

## 7. Foods and NAFLD Amelioration 

The impact of the foods is still controversial due to the limited number of large clinical trials, and the different amounts consumed by the population. 

### 7.1. Nuts

Nuts and seeds are composed of a unique blend of fatty acids and bioactive compounds, such as monounsaturated fatty acids and polyunsaturated fatty acids, vegetable protein, fibre, minerals, vitamins, tocopherols, phytosterols, and polyphenols [80]. All dried fruits have a similar composition in proteins (10–25%), sugars (5–20%), and lipids (50–60%; from 0.51 to 0.73 g/g of fruit), except for chestnuts that are richer in carbohydrates than in fat, and they do not provide cholesterol. Nuts rich in monounsaturated fatty acids, especially oleic acid, are hazelnuts, almonds, and pistachios. Nuts rich in polyunsaturated fatty acids, especially linoleic acid, are walnuts and sunflower seeds. Walnuts are rich in alpha-linolenic acid [80]. Regular consumption of walnuts improves the lipid profile and decreases the incidence of obesity, type 2 diabetes mellitus (T2DM), and hypertension together with MetS. Their consumption is inversely associated with the presence of hepatic transaminases (gamma-glutamyl transferase (GGT), alanine aminotransferase (ALT), and aspartate aminotransferase (AST). Low consumption of nuts and seeds is associated with an increased risk of developing NAFLD in men. A study with 4655 subjects concluded that daily nut consumption is inversely related to advanced fibrosis in NALFD patients [81]. Despite their high energy contents, nut consumption is not associated with weight gain [82]. Nuts improve lipid profile, liver disease, and inflammation, making them useful in a potential treatment for NAFLD [83].

### 7.2. Extra Virgin Olive Oil (EVOO)

Olive oil contains more than 30 phenolic compounds [84]. It is predominantly comprised of MUFAs (70–80%), such as oleic acid (OA; C18:1 *n*-9). It has low amounts of linoleic acid ALA (up to 20%) and palmitic acid (up to 20%) [83]. It contains α-tocopherol (5,7,8-trimethyltocol), the most active in vivo form of vitamin E and the most abundant in nature [85]. Extra virgin olive oil (EVOO) is obtained directly from the ripe fruit by physical means (first pressing or centrifugation), and it is the only one that is consumed unrefined [83]. The beneficial effects of EVOO are widely shown. A randomized controlled trial demonstrated that 10 g of EVOO in a MedDiet context decreased postprandial blood glucose levels and low-density lipoprotein LDL and increased insulin level and glucagon-like peptide-1 (GLP-1) [86,87]. In a human study using EVOO as a source of MUFAs to treat patients with T2DM, a reduction in liver fat (39%) was observed [86]. The consumption of EVOO can alleviate the degree of fatty liver in patients treated with a hypocaloric diet [88]. However, the results should be considered as the effect of other components and the hypocaloric diet. A review of EVOO and its liver-protective effects exposed several relevant molecular effects, such as the induction of cellular antioxidant responses, the prevention of inflammatory responses, and the prevention of the endoplasmic reticulum stress, autophagy, and lipogenic responses [89]. 

### 7.3. Fish

Omega-3 fatty acids are long chains of polyunsaturated fatty acid (PUFAs). Since fish can synthesize omega-3 fatty acids from ingestion of marine plants [84], omega-3 are mostly found in seafood. Marine omega-3 includes eicosapentaenoic acid (EPA), docosahexaenoic acid (DHA), and docosapentaenoic acid [85]. Deep-water fish (tuna, salmon, mackerel, herring, and sardines, globally called “oily fish”) showed the highest EPA and DHA concentrations, because they store lipids in the flesh, contrary to lean species that store them in the liver (cod) and have lower levels of EPA and DHA [90]. By means of several mechanisms, fish oil omega-3 PUFAs decrease lipid accumulation and hepatic enzyme levels, increase insulin sensitivity, and show anti-inflammatory effects [84,91]. In contrast, thrombotic and inflammatory events, such as cardiovascular disease, cancer, and inflammatory and autoimmune illnesses, are associated with high intakes of omega-6 PUFAs. In the Western diet, an omega-6/omega-3 (proinflammatory: anti-inflammatory) ratio of 15:1 has been reported [92]. Thus, the increase in omega-6/omega-3 is implicated in the development of hepatic steatosis and then NAFLD/NASH [91]. A meta-analysis of the efficacy of omega-3 supplementation in people with NAFLD/NASH during a median duration of 6 months showed that omega-3 polyunsaturated fatty acid supplementation reduced liver fat measured by ultrasound, magnetic resonance imaging, or biopsy and improved liver enzymes (AST and ALT) [88]. The depletion of omega-3 fatty acids is correlated with the development of hepatic steatosis and subsequently NAFLD, NASH, and fibrosis [91]. A study in Japan investigated the dietary intake, abdominal fat, and biochemical data of non-obese and obese NAFLD patients, finding that dietary cholesterol was abundant as well as dietary PUFAs that had lower levels in obese patients and healthy controls, concluding that the altered cholesterol and PUFA intake may be associated with NAFLD in non-obese patients [26]. 

### 7.4. Dietary Fibre 

Poor fibre consumption may impair NAFLD; however, the exact mechanism is unclear yet [93,94]. Since some fibres are prebiotic, high-fibre diets and whole grains have the potential to influence positively the composition of the gut microbiota, which may be relevant to the hepatic-gut axis in NAFLD progression [95]. The gross intestinal bacteria ferment fibre into short-chain fatty acids, such as butyrate, propionate, and acetate, which delays the progression of NAFLD [96]. Poor fibre intake is common in NAFLD and NASH [92]. The consumption dietary fibres, especially those derived from whole grains, are beneficial to reduce comorbidities associated with MetS and NAFLD. Moreover, to reduce hepatic fat, fibres derived from whole grains may also reduce inflammation [97]. 

### 7.5. Legumes

Legumes provide essential nutrients such as vegetable proteins, fibre, minerals, oligosaccharides, phytochemicals, and other bioactive substances such as saponins and polyphenols [98]. There are studies reporting the benefits of legumes. A study among Tehrani adults investigated the relationship between legume intake and a risk of NAFLD and concluded that a high total intake of legumes (beans, lentils, and peas) was significantly associated with a low risk of NAFLD [99]. A significant inverse relationship between legume intake and a risk of cardiovascular disease was also reported in a meta-analysis [100]. Dietary intake of non-soy legumes (lentils, beans, peas, and chickpeas) was associated with high-quality diet, low fat intake, high intake of bioactive compounds and vegetable proteins, small waist circumference, and low serum cholesterol and blood pressure [99,100]. Legumes lowered blood lipids by increasing the excretion of fats and steroids through the faeces and the formation of short-chain fatty acids in the colon [101].

### 7.6. Fruits and Vegetables

The mechanism underlying the beneficial effects of vegetable and fruit consumption on the NAFLD risk is fully known; however, it has been explained by its reduced energy density after their addition to the diet, as well as the antioxidant activity of polyphenols and carotenoids contents in vegetables and fruits [102]. A cross-sectional study in overweight Latino youth participants concluded that the consumption of non-starchy vegetables was associated with positive metabolic outcomes, including low visceral and liver fat, and increased insulin sensitivity, even when consumed small amounts [103]. A prospective cohort study on a large Korean population sample [102] showed that vegetable and fruit intakes were individually associated with low incidence of NAFLD in women. The total vegetable and fruit intake was related to low incidence of NAFLD in both men and women after a 4.2-year follow-up. A cross-sectional study in Japan showed no significant association between fruit or vegetable intake and NAFLD [104]. It has been pointed out that added fructose, and not natural fructose as being in vegetables and fruits, is the main contributor to the NAFLD development and progression. Fruit and vegetable fructose has no adverse effects on NAFLD, and protects against NAFLD and other related diseases, such as T2DM [105].

### 7.7. Dairy Products

It has been suggested that dairy products prevent NAFLD due to the contents of whey protein by reducing weight and fat mass [106]. The protective effects of dairy products against NAFLD were attributed to several mechanisms, some connected to insulin resistance. The prevalence of dairy consumption was found to be negatively correlated with the development of the insulin resistance syndrome in the population-based prospective coronary artery risk development in a young adult study [107]. A meta-analysis study revealed that consumption of dairy products, particularly low-fat, had a positive impact on body weight, waist circumference, and the Homeostatic Model Assessment for Insulin Resistance (HOMA-IR) [108]. Cheese and dairy products are a rich source of so-called bioactive peptides [109]. A study conducted in Korea found that high milk protein intake was significantly and inversely associated with the risk of NAFLD incidence in men and women aged ≥50 years. The consumption of milk and other dairy products could prevent the development of NAFLD [110]. A population-based cohort with 7540 adults evaluated the association between the total intake of different dairy products and fatty liver index (FLI), finding a modest inverse correlation between milk consumption and FLI, but they were not able to examine low-fat and high-fat dairy products separately. Nevertheless, they concluded that drinking milk in moderation, especially low-fat varieties (at least one unit more than 5–6 times a week) could ameliorate NAFLD [111]. Yogurt is a food produced by bacterial fermentation of milk, and its consumption delivers many probiotics to the gastrointestinal tract [112]. A cross-sectional study found that high yogurt consumption was associated in a dose-dependent manner with low prevalence of newly diagnosed NAFLD [113]. A randomized double-blinded study demonstrated that the consumption of yogurt containing *Lactobacillus acidophilus* La5 and *Bifidobacterium lactis* Bb12 for 8 weeks in patients with NAFLD showed lower liver aminotransferases, total cholesterol, and LDL-cholesterol compared to patients consuming conventional yogurt. Another randomized controlled trial with 102 patients assessed the effects of symbiotic yogurt (containing 108 colony-forming units of *Bifidobacterium animalis*/mL and 1.5 g inulin) on NAFLD, concluding that symbiotic yogurt consumption improved the hepatic enzyme concentrations [114]. 

## 8. Other Foods and Nutrients That Ameliorate NAFLD 

### 8.1. Probiotics

Probiotics are intestinal bacteria or yeast with the ability to confer health benefits on the host [94,115]. The most used bacteria are *Bifidobacterium* and *Lactobacillus* strains, which can ameliorate NAFLD [116]. The literature shows that probiotics have therapeutic effects on NAFLD, reducing oxidative and inflammatory liver damage, as well as lowering hepatic triglycerides (TG) and hepatic steatosis [117,118]. Altered gut microbiota is associated with obesity, a risk factor for NAFLD. An increase in the strain *Firmicutes* and a decrease in *Bacteroidetes* were described in obesity [119]. Prebiotics are nutrients used by host microorganisms, and they can modulate the composition of the gut microbiota involved in NAFLD development. In Asian NAFLD patients (*n* = 75) using probiotics and prebiotics, there was a decrease in body weight and amelioration of NAFLD parameters [120]. A review highlighted that NASH patients treated with *Lactobacilli* strains plus *Bifidobacterium bifidum* showed low triglyceride liver contents measured by magnetic resonance imaging, but these effects in NASH should be further tested in larger studies [24]. 

### 8.2. Coffee

Coffee protects against the NAFLD development and decreases the NASH severity. The unsweetened filtered coffee could be a reasonable adjunct to diet and exercise in patients with fatty liver [121]. Coffee contains caffeine, phenols, chlorogenic acids, sugars, organic acids, polysaccharides, and aromatics, among more than thousand compounds. Two decades ago, the protective effect of coffee consumption against cirrhosis was reported [122]. A growing body of evidence suggested the beneficial effects of coffee on NAFLD by means of both a direct effect on the liver and systemic metabolic effects. Lately, a high reduction in the risk of T2DM and cardiovascular disease was demonstrated in coffee drinkers [123,124]. The US National Health and Nutrition Examination Survey 2001–2008 showed that caffeine and plain water intakes were independently related to a low risk of NAFLD, regardless of race, gender, clinical parameters, metabolic syndrome components, and dietary constituents [125]. Patients with NAFLD/NASH showed a reduced risk of liver fibrosis after taking coffee. Two meta-analyses concluded that coffee consumption was associated with a 29% lower risk of NAFLD, 30–39% lower risk of liver fibrosis, and a 39% lower risk of cirrhosis; however, the limitation was the definition of regular coffee consumption, because it changed between studies [126]. One study also assessed regular consumption of filtered coffee, but not espresso, and found that it was independently associated with low fibrosis [127]. It was pointed out that espresso drinkers usually added sugar, which explains the benefits of filtered coffee. Caffeine is perhaps the coffee’s best-known compound [127]. Coffee can protect the liver by increasing PPAR-α-mediated fatty acid oxidation, decreasing collagen deposition, and promoting an overall increase in protective antioxidants [128]. Chlorogenic acid, caffeine, and kahweol exhibit antifibrotic properties by the inhibition of hepatic stellate cell activation via the downregulation of the transforming-growth-factor-β (TGF-β) pathway and by inhibiting the connective-tissue growth factor [129,130]. Regarding the dosage of coffee consumption, a meta-analysis described a nonlinear relationship between a reduced risk of NAFLD and consumption of >3 cups/d [131]. A cross-sectional study, where coffee consumption was assessed for 6 months, showed low odds of liver fibrosis after a daily consumption of >2 cups [132]. Low fibrosis score was shown in people that drank more coffee (>3/day); thus, coffee intake can have beneficial effects on fibrosis progression [133].

Coffee consumption may also reduce the risk of developing hepatocellular carcinoma. Consumers of ≥3 cups/day showed a 44% lower risk of developing hepatocellular carcinoma [134]. High levels of coffee consumption were related to a lower risk of incident hepatocellular carcinoma (HCC) and chronic liver disease (CLD) mortality compared to non-coffee drinkers; the benefits were observed in those who drank 2–3 cups per day, and the benefits were higher for those who drank ≥4 cups per day. Moreover, the inverse associations were similar regardless of the participants’ ethnicity, sex, body mass index, smoking status, alcohol intake, or diabetes status [135]. Interestingly, the benefits of coffee consumption could be extended to the gut microbiota, as the literature reported possible associations such as an increase in *Bifidobacterium* spp. [136,137] and a decrease in *Escherichia coli* and *Clostridium* spp. [137]. Coffee consumption is associated with microbial richness in patients with cirrhosis [115,138]. 

### 8.3. Choline

Choline is an essential group B vitamin obtained from both dietary intake (egg yolks and animal protein) and endogenous synthesis, which is mainly metabolized and stored in the liver [139]. Choline is an essential nutrient, and the central organ responsible for choline metabolism is the liver; hepatosteatosis and death of hepatocytes occur when humans are deprived of choline [140]. In humans, choline deficiency affects NAFLD by inducing irregular phospholipid synthesis, defects in lipoprotein secretion, oxidative damage due to mitochondrial dysfunction, and endoplasmic reticulum stress. The requirement of choline to prevent liver dysfunction is dependent on genetic variations and oestrogen status [140]. Human choline requirements are highly individualized, and biomarkers of choline status are useful to predict the risk of NAFLD [140].

### 8.4. Micronutrients 

Micronutrients are necessary for enzyme function, intermediate metabolism, and the metabolic response to sickness. They include electrolytes, minerals, vitamins, and carotenoids [141]. The excess or lack of micronutrients might exacerbate tissue injury and perturbations of energy homeostasis in NAFLD patients by disrupting the lipid homeostasis and antioxidant pathways. It was suggested that understanding the involvement of micronutrients in NAFLD may help to better understand non-obese NAFLD [142]. The relevant micronutrients involved in NAFLD are zinc, copper, iron, selenium, magnesium, vitamins A, C, D, and E, and carotenoids with the antioxidant, antifibrotic, immunomodulatory, and lipo-protective effects proposed as the mechanisms of the impact of micronutrients on NAFLD [142]. Zinc and copper deficiencies were observed in NAFLD [143,144]. Zinc supplementation showed favourable effects on glycaemic parameters and plasma lipids [145]. However, an excess of iron and selenium may increase the severity of NAFLD [142]. Hepatic iron accumulation in reticule-endothelial cells occurs in NAFLD, and it is associated with the NAFLD pathogenesis, but data are often conflicting [146,147]. Vitamin A, B_3_, B_12_, C, D, and E deficiencies—mostly of a low severity—were linked to NAFLD. Vitamin E is an important antioxidant which was used as treatment, decreasing transaminase levels and liver lobular inflammation, improving liver fibrosis, and reducing steatosis [148]. Vitamin E supplementation is a usual practice in NAFLD patients to diminish high oxidative stress. Nevertheless, vitamin E supplementation could have side effects, including an increase in the risk of several kinds of cancer or haemorrhagic stroke, which are the key factors in reducing its use in the clinical practice [25]. Vitamin D protects against the NAFLD and cardiovascular disease by improving insulin sensitivity, reducing inflammation of the adipose tissue, reducing liver inflammation and fibrosis [149]. Low serum vitamin D levels may result in NAFLD, and the severity and incidence of NAFLD are linked to hypovitaminosis D [150]. A recent study in Chinese population showed that low serum vitamin D level was associated with NAFLD in obese but not lean participants [151]. Carotenoids can be found in abundance in colourful fruits and vegetables, and carotenoid-rich foods may be more effective in preventing NAFLD than those with low carotenoid levels [152]. 

### 8.5. NAFLD, Oxidative Stress, Inflammation, and Mediterranean Diet 

Disturbances in lipid metabolism led to liver lipid accumulation, affecting reactive oxygen species (ROS) genesis. Oxidative stress and inflammation play an important role in the development of chronic diseases, such as NAFLD, and then they could be crucial targets in dietary strategies for disease prevention. It was demonstrated that the severity of NAFLD is related to an increase in oxidative stress and proinflammatory status. Plasma levels of catalase, irisin, interleukin-6, malondialdehyde, and cytokeratin 18 were higher in subjects with more than 17.4% of intrahepatic fat contents measured by magnetic resonance imaging, whereas the resolvin D1 levels were lower [153].

There is evidence that plant-based dietary patterns, such as MedDiet, are associated with low oxidative stress and inflammation, providing useful ways for chronic disease prevention [154]. 

A one-year nutritional intervention (with hypocaloric Mediterranean diet) improved the main NAFLD features in 40–60-year-old patients, decreasing body mass index, intrahepatic fat contents, glycosylated haemoglobin, markers of liver damage (ALT, AST, GGT, and cytokeratin-18), and prooxidant (malondialdehyde, myeloperoxidase, zonulin, and omentin) and proinflammatory status (decrease in dietary inflammatory index, C reactive protein, ectodysplasin-A, interleukin-1ra, and interleukin-6) and improving the antioxidant (catalase plasma levels and gene expression in peripheral blood mononuclear cells) and anti-inflammatory (irisin and resolvin D1) effects. There was also a decrease in the concentration of plasmatic endotoxin, suggesting an improvement in intestinal permeability [155,156,157,158].

Evidence highlighted the role of two-series prostaglandins in NAFLD pathogenesis, decreasing insulin secretion and pancreatic β-cell proliferation, increasing gluconeogenesis, enhancing hepatic lipogenesis, and decreasing triglyceride output, thus establishing negative effects on liver inflammation and NAFLD progression [159].

NADPH oxydase (NOX) enzyme isoforms exert both metabolism and inflammation regulatory effects, decreasing liver fibrosis, triglycerides production, inflammation, steatosis, insulin resistance, and ROS generation and increasing insulin resistance [160]. However, these effects were demonstrated in mice and cellular models, and they need to be proved in humans.

In addition, 8-iso-prostaglandin F_2_α was related to some acute-phase-reactant proteins, providing a biochemical link between lipid peroxidation, inflammation, and genesis of NAFLD and other chronic diseases [161]. Therefore, it can be considered as a useful biomarker of oxidative stress in T2DM and related comorbidities, such as NAFLD [162]. Polyphenols contained in the diet, such as MedDiet, produce changes in biomarkers related to the oxidant/inflammatory status, such as 8-iso-prostaglandin F_2_α [163].

Healthy diet, physical activity, prebiotics, probiotics, and healthy faecal microbiota transplantation are the new therapeutics to prevent hepatic oxidative stress in the future [164].

## 9. Conclusions

**Figure 1:**
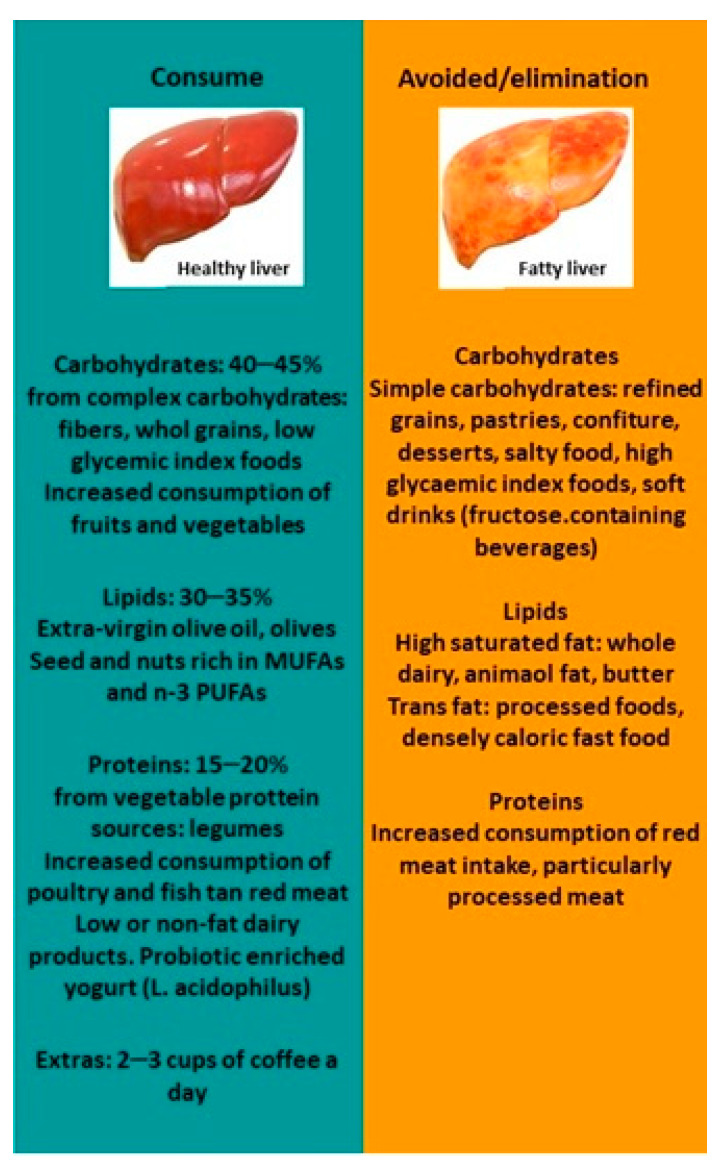
NAFLD diet recommendations. Abbreviations: MUFAs, monounsaturated fatty acids; PUFAs, polyunsaturated fatty acids.

**Figure 2:**
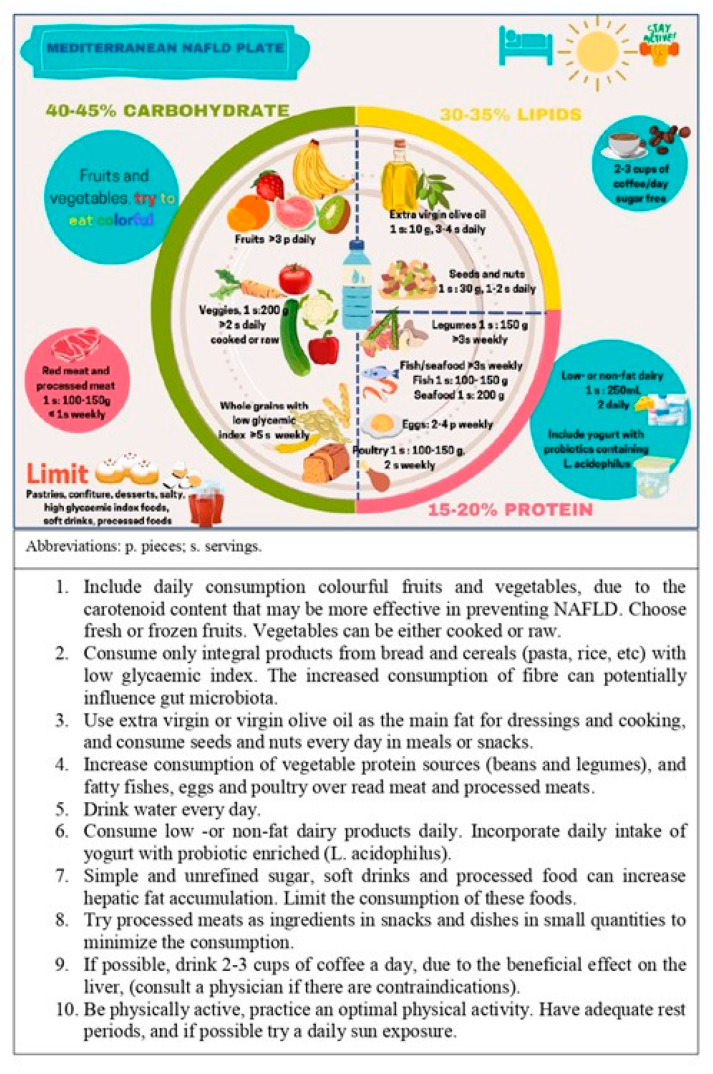
The Mediterranean NAFLD plate. A guideline on daily intake for NAFLD patients.

A high-quality diet could improve liver steatosis. Mediterranean diet together with physical activity and limited sugar intake is a good strategy for managing NAFLD. Specific dietary recommendations are added in this manuscript, and a Mediterranean plate is proposed to ameliorate NAFLD (Figure 1 and Figure 2).

## Figures and Tables

**Table 1:** Recommendations of scientific associations on dietary guidelines for NAFLD.

	Weight Loss and Caloric Restriction	Composition of Diet	Intake of Food or Specific Nutrients	Alcohol Intake
EASL–EASD–EASO [17]	Total weight loss of 7–10%. Energy deficit of 500–1000 kcal.	Low-to-moderate fat and moderate-to-high carbohydrate intake. Low carbohydrate intake or emphasizing MedDiet to reduce liver fat.	Avoid fructose-containing beverages and foods. Coffee drinking is protective in NAFLD; there is no liver-related limitation.	Alcohol intake should be kept below the risk threshold, and total abstinence in NASH
ESPEN [21]	A 7–10% weight loss improves steatosis and liver biochemistry in overweight and obese NAFL-NASH patients; a weight loss of >10% is recommended to reduce liver fibrosis. Hypocaloric diet.	MedDiet to improve steatosis and insulin sensitivity.	Coffee is more likely to benefit than harm.	Abstain
AASLD [20]	Weight loss ≥5% for steatosis improvements and ≥7% for histological improvements. (Decreasing body weight by 5% improves hepatic steatosis and by 7% improves NASH) Decrease caloric intake by 30% or approx. 750–1000 kcal/day.	Prospective trials comparing diets with different macronutrient composition in NAFLD patients are limited by insufficient power as well as pre- and post-treatment histopathology.	Not specified	Not heavy amounts should be consumed; heavy amounts are not specified
APASL [22]	Hypocaloric diet (500–1000 kcal deficit) and gradual weight loss (up to 1 kg/week).	Not enough evidence supporting a dietary approach to solving MAFLD. Dietary plans should encourage low-carbohydrate, low-fat, and Mediterranean-type diets.	Exclusion of beverages high in added fructose. Clear MAFLD-decreased risk and hepatic fibrosis among regular coffee drinkers.	To avoid alcohol and, if it is not possible, to consume its lowest amount. Cutoff value of alcohol intake should be lower than “threshold levels”.

AASLD: American Association for the Study of Liver Diseases; APASL: Asian Pacific Association for the Study of the Liver; EASD: European Association for the Study of Diabetes; EASL: European Association for the Study of the Liver; EASO: European Association for the Study of Obesity; ESPEN: European Society for Clinical Nutrition and Metabolism; MAFLD: metabolic-associated fatty liver disease.

## Data Availability

There are restrictions on the availability of data for this trial, due to the signed consent agreements around data sharing, which only allow access to external researchers for studies following the project purposes. Requestors wishing to access the trial data used in this study can make a request to pep.tur@uib.es.
